# Observation of Efficacy of Internet-Based Chronic Disease Management Model Combined with Modified Therapy of Bushenyiliu Decoction in Treating Patients with Type 2 Diabetes Mellitus and Prostate Cancer and Its Effect on Disease Control Rate

**DOI:** 10.1155/2021/7767186

**Published:** 2021-09-09

**Authors:** Yi Wang, Ankang Yin, Tingting Bian, Xiangyu Zhao, Shijun Zheng, Weihua Hou, Yan Wang, Yuan Liu

**Affiliations:** ^1^Department of General Practice, Affiliated Hospital of Yangzhou University, Yangzhou 225000, Jiangsu Province, China; ^2^Department of Pharmacy, Yangzhou Matemal and Child Care Service Centre, Yangzhou 225000, Jiangsu Province, China; ^3^Department of Education, Affiliated Hospital of Yangzhou University, Yangzhou 225000, Jiangsu Province, China

## Abstract

**Objective:**

To explore the efficacy of Internet-based chronic disease management model combined with the modified therapy of Bushenyiliu decoction in treating patients with type 2 diabetes mellitus (T2DM) and prostate cancer and its effect on disease control rate (DCR).

**Methods:**

120 patients with T2DM and prostate cancer admitted to the Affiliated Hospital of Yangzhou University, Yangzhou First People's Hospital, from February 2019 to February 2020, were retrospectively analyzed and equally divided into the experimental group and the control group according to their admission order. Conventional treatment combined with the modified therapy of Bushenyiliu decoction was performed on all patients for 3 months, and the Internet-based chronic disease management model was adopted for patients in the experimental group additionally, so as to compare their short-term effect, survival time, disease progression, blood glucose indicators, immune function indicators, and type 2 Diabetes Self-Care Scale (2-DSCS) scores.

**Results:**

Compared with the control group, the experimental group obtained significantly higher DCR and objective remission rate (ORR) (*P* < 0.05), higher survival time and disease progression (*P* < 0.001), better blood glucose indicators and immune function indicators (*P* < 0.001), and higher 2-DSCS scores (*P* < 0.001) after treatment.

**Conclusion:**

Combining the Internet-based chronic disease management model with the modified therapy of Bushenyiliu decoction can effectively enhance the self-care ability of patients with T2DM and prostate cancer, improve their blood glucose level, promote their body immunity, and comprehensively optimize the cancer control effect, which should be promoted in practice.

Prostate cancer is an epithelial malignancy that occurs in the prostate with complex pathogenic factors, and patients with prostate cancer often present with abnormal urination, pelvic discomfort, and other symptoms. Its incidence and mortality rates, respectively, rank the 2nd and the 6th of male malignancies, and in European and American countries, the number of patients who die from this disease each year accounts for 6% of deaths from malignant tumors [1–3]. The incidence of prostate cancer in China is lower than the world average, and the disease lethality was found to account for only 1.11% of the mortality from malignant tumors in China in the 2004–2005 sampling survey [4, 5]. However, China has seen the increase in the incidence of prostate cancer in the recent decade, with an annual increase of approximately 12.07% [6], as a result of which prostate cancer is likely to become the most prevalent urological malignancy among Chinese men [7]. Although the incidence of prostate cancer is increasing year by year, its early diagnosis rate has not been significantly improved, and patients are mostly in the metastatic or advanced stage when diagnosed. The role of radical surgery, chemotherapy, and endocrine therapy is limited, and treatment methods such as chemotherapy, like a double-edged sword, will greatly reduce the patients' body immunity, so screening a treatment method that is safer and highly effective has become a research hotspot in the field of prostate cancer treatment.

Recent clinical experiments have confirmed the unique advantages of traditional Chinese medicine (TCM) holism and the therapy with syndrome differentiation in treating the malignant tumors, and both Bushenyiqi decoction and Fuzhengyiliu decoction can improve the functional status of prostate cancer patients [8, 9]. The latest studies have shown that Bushenyiqi decoction can affect the Notch signaling pathway and regulate PC-3 cells [10], which has a precise efficacy and higher safety, indicating that TCM treatment is able to enhance the disease control effect of patients. However, clinical experiments usually focus on the treatment of the prostate and ignore the multiple complications that may exist in patients with prostate cancer, and the influence of different complications on the efficacy of TCM treatment is not clear. Type 2 diabetes mellitus (T2DM) is one of the most common complications of prostate cancer, and there are mixed opinions among academia regarding whether the disease is a risk factor predisposing to prostate cancer, but most of the literature have identified that T2DM is closely related to the prognosis of prostate cancer patients [11]. Bjornsdottir and other scholars suggested that T2DM can increase the mortality rate of prostate cancer patients [12], and the levels of blood glucose and glycosylated hemoglobin (HbA1c) can affect the division and apoptosis of prostate cancer cells, so the control of blood glucose level is the focus of treatment for patients with prostate cancer and T2DM. The World Health Organization (WHO) proposes that chronic disease management is a cosmopolitan conundrum [13], and even in Shanghai, where the level of self-care is high, the rate of blood glucose achievement is still under 10%. Some scholars have shown that the “Internet Plus” chronic disease management model based on mobile communication technology can improve the blood glucose control effect and enhance the patients' treatment compliance [14], which may work in both T2DM and prostate cancer at the same time.

Hence, 120 patients with T2DM and prostate cancer were included in the study to explore the efficacy of combining Internet-based chronic disease management model with the modified therapy of Bushenyiliu decoction in treating patients with T2DM and prostate cancer was explored herein, with the study reported as follows.

## 1. Materials and Methods

### 1.1. Study Design

This retrospective study was conducted in the Affiliated Hospital of Yangzhou University, Yangzhou First People's Hospital, from February 2019 to February 2020, and aimed to explore the efficacy of the combination of Internet-based chronic disease management model and the modified therapy of Bushenyiliu decoction in treating patients with T2DM and prostate cancer and its effect on disease control rate (DCR).

### 1.2. Enrollment of Research Objects

Patients with T2DM and prostate cancer admitted to our hospital from February 2019 to February 2020 were retrospectively analyzed with the following inclusion and exclusion criteria. Inclusion criteria: (1) the patients met the diagnosis criteria for T2DM established by WHO in 1999 [10] and were diagnosed with prostate cancer after pathological examination and imaging examination; (2) the patients were 18 to 76 years old; (3) the patients accepted the insulin treatment and did not accept chemoradiotherapy before enrollment; and (4) the duration of diabetes of the patients was over 1 year. Exclusion criteria: (1) the patients presented mental problems or were unable to communicate with others; (2) the patients had various acute complications and dysfunction of important organs such as heart, brain, and kidney and abnormal hematopoietic function or suffered from malignant tumors; (3) the patients had gestational diabetes mellitus; (4) the patients' Karnofsky score (KPS) was less than 50 points [15]; and (5) the patients' condition was unstable and had the need for discontinuation of glucose lowering medications.

### 1.3. Steps

A total of 120 patients were enrolled in the study and equally divided into the experimental group and the control group according to their admission order. On the day that patients agreed to join the study, the study team collected the sociodemographic data and clinical manifestation data and found that there were no statistical differences in patients' general information between the two groups after analysis (*P* > 0.05); see Table 1; before treatment, the study team measured the patients' blood glucose indicators and immune function indicators and recorded their type 2 Diabetes Self-Care Scale (2-DSCS) scores; 6 weeks and 12 weeks after treatment, the aforesaid indicators were measured again, and the 2-DSCS scores at the 36th week of follow-up visit were collected; and after 1 year of follow-up visit, the patients' survival time and disease progression were calculated.

### 1.4. Moral Consideration

The study met the principle of *World Medical Association Declaration of Helsinki* [16] and was approved by the ethics committee of the Affiliated Hospital of Yangzhou University, Yangzhou First People's Hospital. After enrollment, the study team explained the study purpose, meaning, content, and confidentiality to the patients and asked them to sign the informed consent.

### 1.5. Criteria of Quitting the Experiment

If the patients had the following situations and were determined as unsuitable to continuously accept the study judged by the study team, their case records were all retained for full data analysis: (1) those who experienced adverse events or serious adverse events; (2) those with condition worsened, etc. during the experiment; (3) those who developed certain severe comorbidities or complications; and (4) those who were unwilling to continue the clinical trial and requested for quitting the experiment to the study team.

### 1.6. Methods

All patients accepted the conventional treatment combined with the modified therapy of Bushenyiliu decoction for three months. (1) ① Prostate cancer treatment: the total androgen deprivation therapy was adopted, intermittent endocrine blockade was performed after maximum androgen blockade, bilateral orchidectomy was conducted, and 250 mg of flutamide (manufactured: Jiangsu Tasly Diyi Pharmaceutical Co., Ltd.; NMPA approval no. H19990144) was taken after three meals every day; if the patients presented painful bone metastases, zoledronic acid (manufactured: Yangtze River Pharmaceutical (Group) Co., Ltd.; NMPA approval no. H20041975) was added; the chemotherapy project was intravenous infusion of 75 mg/m^2^ of docetaxel (manufactured: Yangtze River Pharmaceutical (Group) Co., Ltd.; NMPA approval no. H20058719) once every three weeks and orally taking 5 mg of prednisone (manufactured: Beijing Continent Pharmaceutical Co., Ltd.; NMPA approval no. H20058375) twice a day. ② T2DM treatment: the patients orally took 500 mg of metformin (manufactured: North China Pharmaceutical Co., Ltd.; NMPA approval no. H20113492) and 50 mg of vildagliptin (manufactured: Novartis Pharma Stein AG; NMPA approval no. J20180055) twice every day. (2) Bushenyiliu decoction: ① the formula included epimedium herb, malaytea scurfpea fruit, glossy privet fruit, phellodendri amurensis cortex, mongolian milkvetch root, tangshen, largehead atractylodes rhizome, indian bread exodermis, coix seed, hedyotis, and hawthorn fruit (30 g each) and common yam rhizome, cibot rhizome, himalayan teasel root, air potato, Chinese angelica, and snakegourd root (10 g each); the herbs were decocted with warm water and taken once every day. ② The formula was modified according to condition. 2-3 pieces of fresh ginger were added in case of vomiting; 10 g of sanqi and 10 g of ophicalcite were added in case of internal blockade of static blood; 10 g of two-toothed achyranthes root and 10 g of Chinese taxillus herb were added in case of yin deficiency of liver and kidney; and 10 g of talc was added in case of internal exuberance of damp-heat.

The Internet-based chronic disease management model was adopted for patients in the experimental group additionally with the specific steps as follows. (1) Designing the chronic disease online management miniprogram: The Wechat miniprogram was developed by personnel from a software development company jointly with our hospital, which included the backstage management side and the patient side, being used by the medical workers and patients as well as their family members, respectively. (2) Building a chronic disease online management team: The team that included 2 endocrinologists, 2 prostate cancer physicians, 4 diabetes specialist nurses, 4 prostate cancer specialist nurses, and 2 nutritionists established a one-to-many management model with the patients, accepted the miniprogram training before the study, joined the theoretical examination and operational examination after training, and obtained the access to the backstage management side after getting the qualification certificate. (3) On the day of patients being enrolled in the experimental group, rehabilitation plans were proposed according to their actual condition, diagnosis, treatment, diet, and exercise plans with individual differences were made, one-to-one training was conducted aiming at the chemotherapy and insulin injection for patients, and then the team helped the patients to join the Wechat group, follow the Wechat miniprogram, and use the miniprogram correctly, to improve the utilization rate of the management platform. (4) Chronic disease online management miniprogram module: ① Electronic health records: The patients filled in the electronic health record that was updated once every two weeks with their basic information including blood pressure, blood glucose, weight, cancer stage, family history, and prior history, the system intelligently identified the health status of the patients and emphatically monitored their blood glucose values, hepatic and kidney function, etc., and then the physicians of the platform reminded the patients of physical examination and urged the patients to upload data, so as to dynamically monitor their condition changes in real time; the miniprogram could analyze the data sent by patients each time and reminded the patients with the risk of the deterioration of disease and at the same time sent message to the physicians of the platform; then the physicians gave proper advice and guidance to patients so that they could understand their own condition changes. ② Health consultation: the medical workers established the disease symptoms database for patients to do the self-service enquiry and find correspondent medical advice; in case the database could not satisfy the patient needs, the patients could enter the interface of physician service and raise questions, which the physicians needed to reply within 2 days. ③ Lifestyle assessment: it included the patients' dietary habits, exercise habits, and occupational habits, and by referring to the functional software such as Boohee, the miniprogram could help the patients record their daily diet and exercise and self-screen their health status with the authoritative scales confirmed by international literature and automatically mark the patients with poor scale scores as the key intervention object; then the physicians pointed out their health risks in life and provided reasonable advice and guidance. ④ Sign-in: the sign-in module was based on the calendar and recorded the dates of logging in the miniprogram, return visits, and blood pressure recording, etc. of patients; the miniprogram pushed the sign-in reminder at 9 pm every day to the patients who did not record the information on time. ⑤ Health education: the knowledge resources about disease prevention and treatment were integrated according to the characteristics of diabetes and prostate cancer; information in popular and easy-to-understand language was screened from newspapers and scientific articles and pushed to Wechat in the form of articles, pictures, and short videos, so as to promote the patients' passive reading volume and increase their health knowledge. (5) All patients constantly recorded their data on the miniprogram and accepted telephone follow-up and clinical follow-up for a year.

### 1.7. Observation Criteria

1. General information: the general information extraction forms were established by the patients themselves, including the in-patient number, name, age, duration of prostate cancer, duration of T2DM, blood pressure, fasting blood glucose (FBG) value, cancer metastasis status, level of total prostatic specific antigen (T-PSA), pathological type, Karnofsky score (KPS), place of residence, monthly income, life habits, and educational degree.
2. Short-term efficacy: the patients' condition was assessed according to the Response Evaluation Criteria in Solid Tumors (RECIST) [17] established by WHO in 2000, which classified the condition as complete response (CR, disappearance of all lesions, no new lesions, and recovery of tumor markers for over one month), partial response (PR, ≥ 30% decrease in SLD (the sum of the longest diameters) of the target lesion for over one month), stable disease (SD, no PR, no PD), and progressive disease (PD, ≥ 20% increase in SLD or new lesions). The objective remission rate (ORR, CR + PR) and disease control rate (DCR, CR + PR + SD) were used to compare the treatment effect of patients.
3. Survival time and disease progression: the survival time and disease progression of patients in both groups were calculated.
4. Blood glucose indexes: before treatment (*T*_1_), 6 weeks after treatment (*T*_2_) and 12 weeks after treatment (*T*_3_), 5 ml of fasting vein blood was drawn from the patients in the morning to separate the supernatant under 3,000 r/min after letting it stand for 0.5 h, the FBG values were measured by the glucose oxidase method (Cobase 411 fully automatic biochemical analyzer and original supporting agents; NMPA (I) 20113402843), and the glycosylated hemoglobin (HbA1c) was measured by the cation-exchange high-performance liquid chromatography (EC-HPLC) method (with the machine and original supporting agents made by Tai'an City Kang Yu Medical Instrument Co., Ltd.; Shandong MPA (I) 20142400498) to calculate the standard deviation and absolute deviation of blood glucose.
5. Immune function indicators: at *T*_1_, *T*_2_, and *T*_3_, 5 ml of fasting vein blood was drawn from the patients in the morning, and their levels of T lymphocyte subsets, including CD3^+^, CD4^+^, and CD8^+^, were detected and their CD4^+^/CD8^+^ values were calculated with the flow cytometer (FCM) (manufactured: Acea Bio (Hangzhou) Co., Ltd.; Zhejiang MPA Certified No. 20142400581).
6. 2-DSCS scores: the 2-DSCS [18] was made by Wang Jingxuan et al. and the 5-point Likert scale was adopted. The 2-DSCS included the aspects of dietary self-management, regular exercise, medication compliance, blood glucose monitoring, feet care, and prevention and treatment of hyperglycemia and hypoglycemia and a total of 26 items. Each item was evaluated on a scale of 0–5 points, respectively indicating never finished, seldom finished, sometimes finished, often finished, and completely finished. The range of the total score of 2-DSCS was 26–130 points, with higher scores indicating better self-care execution of T2DM patients. The 2-DSCS scores of patients were compared between the two groups at *T*_3_ and the 36th week of follow-up.

### 1.8. Statistical Processing

In this study, the data processing software was SPSS20.0, the picture drawing software was GraphPad Prism 7 (GraphPad Software, San Diego, USA), items included were enumeration data and measurement data, methods used were X^2^ test and *t*-test, and differences were considered statistically significant at *P* < 0.05.

## 2. Results

### 2.1. Comparison of Patients' General Information

No statistical differences were presented when comparing the patients' general information between the two groups (*P* > 0.05); see Table 1.

### 2.2. Comparison of Patients' Short-Term Efficacy

The experimental group obtained significantly higher ORR and DCR than the control group (*P* < 0.05); see Table 2.

### 2.3. Comparison of Patients' Survival Time and Disease Progression

**Figure 1:**
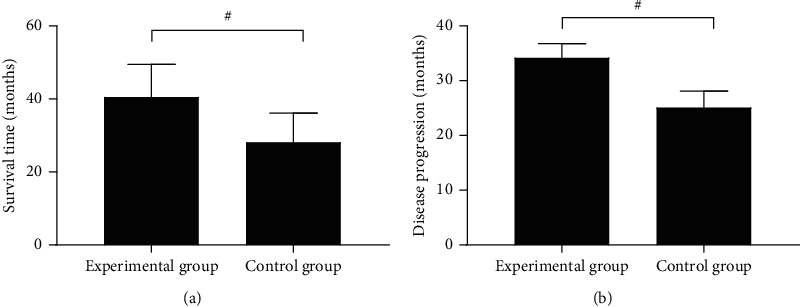
Comparison of patients' survival time and disease progression (‾*x* ± *s*, months). The horizontal axes from left to right denote the experimental group and the control group, and # denotes *P* < 0.001. In Figure 1(a), the vertical axis indicate the survival time, of which the experimental group was significantly higher than the control group (40.56 ± 8.96 vs. 28.12 ± 7.98, *P* < 0.001). In Figure 1(b), the vertical axis indicate the disease progression, of which the experimental group was significantly higher than the control group (34.21 ± 2.56 vs. 25.12 ± 2.98, *P* < 0.001).

The survival time and disease progression were significantly higher in the experimental group than in the control group (*P* < 0.001); see Figure 1.

### 2.4. Comparison of Patients' Blood Glucose Indicators

**Figure 2:**
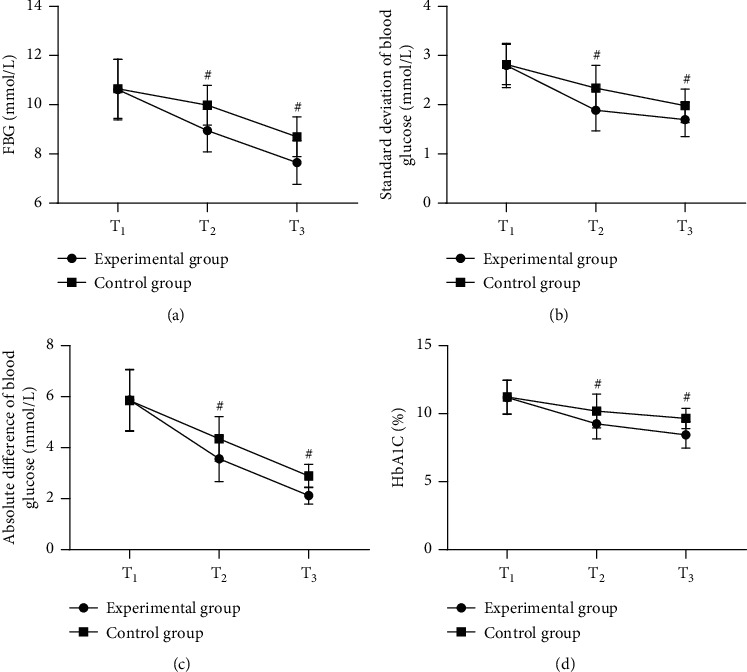
Comparison of patients' blood glucose indicators (‾*x* ± *s*). The horizontal axes from left to right denoted *T*_1_, *T*_2_, and *T*_3_, the lines with dots denote group A, and the lines with blocks denote group B; and # denote *P* < 0.001. In Figure 2(a), the vertical axis indicate the FBG values; at *T*_1_, the FBG values of the two groups were not statistically different (10.62 ± 1.23 vs. 10.65 ± 1.20, *P* > 0.05); and at *T*_2_ and *T*_3_, the FBG values were significantly lower in the experimental group than in the control group (8.95 ± 0.87 vs. 9.98 ± 0.81, 7.65 ± 0.89 vs. 8.70 ± 0.80, *P* < 0.001). In Figure 2(b), the vertical axis indicate the standard deviation of blood glucose; at *T*_1_, the standard deviations of blood glucose of the two groups were not statistically different (2.80 ± 0.45 vs. 2.82 ± 0.41, *P* > 0.05); and at *T*_2_ and *T*_3_, the standard deviations of blood glucose were significantly lower in the experimental group than in the control group (1.89 ± 0.42 vs. 2.34 ± 0.46, 1.70 ± 0.35 vs. 1.98 ± 0.34, *P* < 0.001). In Figure 2(c), the vertical axis indicate the absolute deviation of blood glucose; at *T*_1_, the absolute deviations of blood glucose of the two groups were not statistically different (5.87 ± 1.20 vs. 5.85 ± 1.21, *P* > 0.05); and at *T*_2_ and *T*_3_, the absolute deviations of blood glucose were significantly lower in the experimental group than in the control group (3.56 ± 0.89 vs. 4.35 ± 0.87, 2.12 ± 0.34 vs. 2.89 ± 0.45, *P* < 0.001). In Figure 2(d), the vertical axis indicate the HbA1c level; at *T*_1_, the HbA1c levels of the two groups were not statistically different (11.20 ± 1.26 vs. 11.23 ± 1.24, *P* > 0.05); and at *T*_2_ and *T*_3_, the HbA1c levels were significantly lower in the experimental group than in the control group (9.26 ± 1.10 vs. 10.20 ± 1.24, 8.45 ± 0.98 vs. 9.65 ± 0.74, *P* < 0.001).

After treatment, the experimental group achieved remarkably better blood glucose indicators than the control group (*P* < 0.001); see Figure 2.

### 2.5. Comparison of Patients' Immune Function Indicators

**Figure 3:**
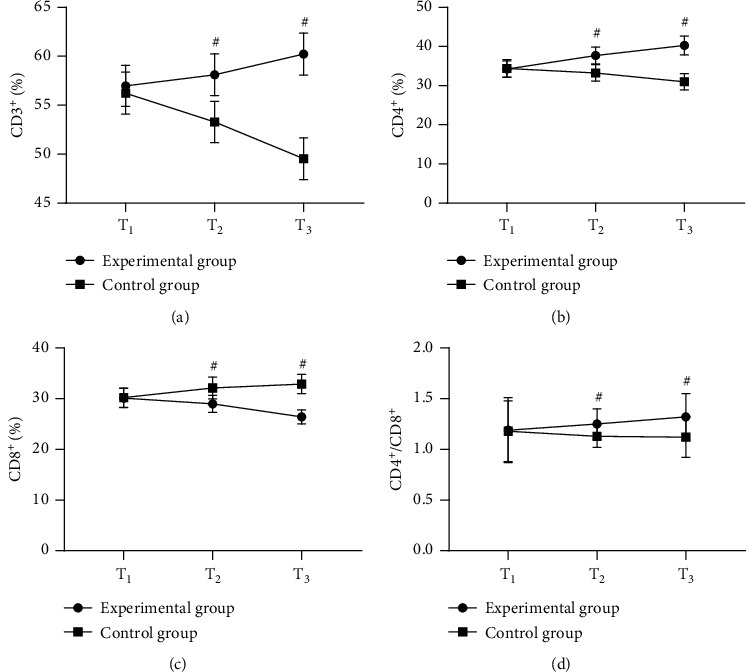
Comparison of patients' immune function indicators (‾*x* ± *s*). The horizontal axes from left to right denote *T*_1_, *T*_2_, and *T*_3_, the lines with dots denote group A, and the lines with blocks denote group B; and # denote *P* < 0.001. In Figure 3(a), the vertical axis indicate the CD3^+^ cells; at *T*_1_, the CD3^+^ cells of the two groups were not statistically different (56.98 ± 2.10 vs. 56.23 ± 2.14, *P* > 0.05); and at *T*_2_ and *T*_3_, the CD3^+^ cells were significantly higher in the experimental group than in the control group (58.11 ± 2.14 vs. 53.29 ± 2.10, 60.23 ± 2.15 vs. 49.54 ± 2.13, *P* < 0.001). In Figure 3(b), the vertical axis indicate the CD4^+^ cells; at *T*_1_, the CD4^+^ cells of the two groups were not statistically different (34.21 ± 2.10 vs. 34.41 ± 2.23, *P* > 0.05); and at *T*_2_ and *T*_3_, the CD4^+^ cells were significantly higher in the experimental group than in the control group (37.68 ± 2.12 vs. 33.24 ± 2.11, 40.25 ± 2.41 vs. 30.98 ± 2.10, *P* < 0.001). In Figure 3(c), the vertical axis indicate the CD8^+^ cells; at *T*_1_, the CD8^+^ cells of the two groups were not statistically different (30.13 ± 1.89 vs. 30.20 ± 1.87, *P* > 0.05); and at *T*_2_ and *T*_3_, the CD8^+^ cells were significantly lower in the experimental group than in the control group (28.98 ± 1.68 vs. 32.10 ± 2.14, 26.41 ± 1.41 vs. 32.89 ± 1.88, *P* < 0.001). In Figure 3(d), the vertical axis indicate the CD4^+^/CD8^+^ ratio; at *T*_1_, the CD4^+^/CD8^+^ ratios of the two groups were not statistically different (1.19 ± 0.32 vs. 1.18 ± 0.30, *P* > 0.05); and at *T*_2_ and *T*_3_, the CD4^+^/CD8^+^ ratios were significantly higher in the experimental group than in the control group (1.25 ± 0.15 vs. 1.13 ± 0.11, 1.32 ± 0.23 vs. 1.12 ± 0.20, *P* < 0.001).

After treatment, the immune function indicators were remarkably better in the experimental group than in the control group (*P* < 0.001); see Figure 3.

### 2.6. Comparison of Patients' 2-DSCS Scores

After treatment, the 2-DSCS scores were remarkably higher in the experimental group than in the control group (*P* < 0.001); see Table 3.

## 3. Discussion

Diabetes mellitus is the metabolic syndrome characterized by chronic increases in blood glucose levels. China has more than 100 million cases of diabetes mellitus, making the incidence of the disease ranking first in the world [19]. 90% of diabetes is T2DM, and recent epidemiological reports have confirmed that the disease is closely related to malignancy due to the facts that malignancy occurs in T2DM patients at a significantly increased risk than the general population and that there is an important relationship between patient prognosis and glycemic control [20]. Bjornsdottir and other scholars pointed out that T2DM increased mortality in patients with prostate cancer, with implicated factors including insulin-like growth factor I, insulin, blood glucose, and HbA1c. Insulin-like growth factor I and insulin are able to promote tumor development, long-term maintenance of high blood glucose levels increases the risk of prostate cancer recurrence, and prostate cancer patients with T2DM undergoing radical prostatectomy have a 50% increase in recurrence rate compared with prostate cancer patients with normal blood glucose values [21], suggesting that blood glucose levels may serve as a predictor of postoperative recurrence in T2DM patients with prostate cancer. Therefore, it is extremely important to strengthen the glycemic control of patients. However, the universal glycemic control rate across China is low, which is less than 10% even in Shanghai, where medical conditions and scientific research conditions are more advanced, and the HbA1c control rate of Grade III Level A hospitals in Beijing is only 37.8% [22], showing that diabetes management is still an important problem in the management of chronic diseases in China.

Based on the Guiding Opinions on Actively Promoting the “Internet Plus” Action Plan released by the State Council, Han and other scholars proposed the “Internet Plus” chronic management model, aiming to make the most of the convenience of the Internet, improve the use efficiency of medical resources, reduce the medical cost of chronic disease management, and enable diabetic patients to obtain better medical services [23]. The results of Han et al. showed that the “Internet Plus” chronic management mode could improve metabolic parameters in T2DM patients, with significantly higher self-care scale scores in patients with diabetes than in those in the control group (*P* < 0.001), which was similar to the results herein, in which the experimental group had significantly higher posttreatment 2-DSCS scores than the control group (*P* < 0.001), demonstrating that this chronic disease management model could achieve seamless docking of home care and hospital resources, promote the information sharing between doctors and patients, and fully address the decreasing regulatory efforts when patients were at home. To improve the efficiency of “Internet Plus” chronic management mode, Wechat miniprogram, with the advantages of real-time operation and convenience and the utilization better than computer-based platforms, was used as a tool in this study. For patients with prostate cancer, mobile phones are also a more practical tool. In this study, after regular entry of personal information, the patient's T2DM data and prostate cancer data were under dynamic regulation, so the improved glycemic control rate and posttreatment blood glucose indicators of the experimental group were significantly better than those of the control group (*P* < 0.001).

Improvement in blood glucose indicators is key to the consolidation of therapeutic effects in prostate cancer. Hyperglycemia inhibits docetaxe-induced apoptosis of prostate cancer cells, while there is a positive correlation between HbA1c and metastasis of prostate cancer, so the numerical reduction of both is beneficial for Bushenyiliu decoction to exert therapeutic effects. In Bushenyiliu decoction, epimedium herb, malaytea scurfpea fruit, glossy privet fruit, phellodendri amurensis cortex, and other herbs can inhibit proliferation and induce apoptosis of PC-3 cells and are conducive to slowing down the progression of prostate cancer, which has been proved in the in vitro study [24]; in addition, mongolian milkvetch root and tangshen have the effect of supplementing spleen to nourish lung, while largehead atractylodes rhizome can invigorate stomach, and fried orange fruit plays a role in regulating functional activities of vital energy and helping the patients to regulate their qi and blood; from the perspective of modern pharmacology, although tangshen cannot accelerate the rate of tumor cells apoptosis, it can promote the cellular metabolism in malignant tumor host, and there is a counter-promotion effect of largehead atractylodes rhizome, which can selectively inhibit the cell subset of malignant tumor responsible for metastasis; mongolian milkvetch root is able to accelerate protein synthesis, promote energy metabolism, and induce the generation of interleukin, the anticancer factor in the body, tuckahoe can affect the macrophage and lymphocyte, and coixenolide in the coix seed can fully enhance body immunity. Hence, the immune function indicators of the experimental group were significantly better than those of the control group after treatment (*P* < 0.001).

In conclusion, with the combined action of multiple factors, the DCR and ORR of the experimental group were significantly higher than those of the control group (*P* < 0.05), and so were the survival time and disease progression (*P* < 0.001), indicating that the Internet-based chronic disease management mode combined with the modified therapy of Bushenyiliu decoction can effectively enhance the self-care ability of patients with T2DM and prostate cancer, improve their blood glucose level, boost their body immunity, and comprehensively optimize the cancer control effect, which should be promoted in practice.

## Figures and Tables

**Table 1:** Comparison of patients' general information.

Group	Experimental group (*n* = 60)	Control group (*n* = 60)	X^2^/*t*	*P*
Age (years)				
Range	52–76	54–74		
Mean age	68.21 ± 2.65	68.26 ± 2.54	0.106	0.916

Duration of prostate cancer (months)				
Range	2–48	2–49		
Mean disease duration	30.24 ± 2.16	30.56 ± 2.15	0.813	0.418

Duration of T2DM (years)				
Range	1–12	1–11		
Mean disease duration	5.23 ± 1.22	5.26 ± 1.23	0.134	0.894

Blood pressure (mmHg)				
Diastolic blood pressure	78.65 ± 5.65	79.10 ± 5.10	0.458	0.648
Systolic blood pressure	132.65 ± 5.20	132.68 ± 5.23	0.032	0.975
FBG value (mmol)	10.62 ± 1.23	10.65 ± 1.20	0.135	0.893

Metastasis status				
Brain metastasis	3	4	0.152	0.697
Lung metastasis	4	5	0.120	0.729
Liver metastasis	4	3	0.152	0.697
Bone metastasis	6	8	0.323	0.570

T-PSA (*μ*g/L)				
Range	50–456	52–470		
Mean T-PSA	220.25 ± 1.26	220.68 ± 1.25	1.877	0.063

Pathological type				
Adenocarcinoma	48	50	0.223	0.637
Squamous cell carcinoma	8	6	0.323	0.570
Undifferentiated carcinoma	4	4	0.000	1.000
KPS	62.98 ± 2.65	62.35 ± 2.41	1.362	0.176

Place of residence			0.134	0.715
Urban area	32	30		
Rural area	28	30		

Monthly income (yuan)			0.137	0.711
≥4000	24	26		
<4000	36	34		

Lift habits				
Smoking history	35	36	0.035	0.853
Drinking history	38	34	0.556	0.456

Educational degree			0.134	0.715
Senior high school and below	30	28		
College and above	30	32		

**Table 2:** Comparison of patients' overall efficacy (*n*(%)).

Group	CR	PR	SD	PD	ORR	DCR
Experimental group	18 (30.0)	30 (50.0)	6 (10.0)	6 (10.0)	48 (80.0)	54 (90.0)
Control group	10 (16.7)	20 (33.3)	12 (20.0)	18 (30.0)	30 (50.0)	42 (70.0)
*X* ^2^	2.981	3.429	2.353	7.500	11.868	7.500
*P*	0.084	0.064	0.125	0.006	0.001	0.006

**Table 3:** Comparison of patients' 2-DSCS scores (‾*x* ± *s*, points).

Item		Experimental group	Control group	*t*	*P*
Dietary self-management	*T*_3_	23.68 ± 2.10	18.21 ± 1.52	16.344	<0.001
	36 weeks of follow-up	25.14 ± 2.33	15.42 ± 1.24	28.526	<0.001

Regular exercise	*T*_3_	15.20 ± 1.20	10.10 ± 1.11	24.167	<0.001
	36 weeks of follow-up	17.23 ± 1.20	11.98 ± 1.23	23.665	<0.001

Medication compliance	*T*_3_	12.10 ± 0.89	10.20 ± 1.23	9.694	<0.001
	36 weeks of follow-up	14.12 ± 0.21	9.84 ± 0.87	37.043	<0.001

Blood glucose monitoring	*T*_3_	12.41 ± 1.52	10.95 ± 1.22	5.802	<0.001
	36 weeks of follow-up	14.23 ± 1.51	9.98 ± 0.87	18.890	<0.001

Feet care	*T*_3_	18.24 ± 1.65	16.55 ± 1.42	6.013	<0.001
	36 weeks of follow-up	20.98 ± 1.23	14.32 ± 1.58	25.764	<0.001

Prevention and treatment of hyperglycemia and hypoglycemia	*T*_3_	13.20 ± 1.52	11.87 ± 1.10	5.491	<0.001
	36 weeks of follow-up	15.01 ± 1.20	10.54 ± 1.02	21.985	<0.001

## Data Availability

Data to support the findings of this study are available upon reasonable request to the corresponding author.
